# Assessment of denosumab treatment effects and imaging response in patients with giant cell tumor of bone

**DOI:** 10.1186/s12957-018-1478-3

**Published:** 2018-09-19

**Authors:** Jacob Engellau, Leanne Seeger, Robert Grimer, Robert Henshaw, Hans Gelderblom, Edwin Choy, Sant Chawla, Peter Reichardt, Michael O’Neal, Amy Feng, Ira Jacobs, Zachary J. Roberts, Ada Braun, Bruce A. Bach

**Affiliations:** 1 Department of Oncology, Lund University Hospital, SE-221 85 Lund, Sweden; 2 UCLA Health System, Los Angeles, CA USA; 3 Royal Orthopaedic Hospital, Birmingham, UK; 4 MedStar Georgetown Orthopedic Institute, Washington, DC USA; 5 Leiden University Medical Center, Leiden, Netherlands; 6 Massachusetts General Hospital, Boston, MA USA; 7 Sarcoma Oncology Center, Santa Monica, CA USA; 8 HELIOS Klinikum Berlin-Buch, Berlin, Germany; 9 CoreLab Partners (now known as Bioclinica), Princeton, NJ USA; 10 Amgen Inc., Thousand Oaks, CA USA; 11 Atara Biotherapeutics, Inc, San Francisco, CA USA; 12 Pfizer Inc., New York, NY USA; 13 Kite Pharma, Inc., Santa Monica, CA USA; 14 Pharmacyclics, Sunnyvale, CA USA; 15 AbbVie, Redwood City, CA USA

**Keywords:** Giant cell tumor of bone, Denosumab, RANKL, Objective tumor response

## Abstract

**Background:**

Denosumab has been shown to reduce tumor size and progression, reform mineralized bone, and increase intralesional bone density in patients with giant cell tumor of bone (GCTB); however, radiologic assessment of tumors in bone is challenging. The study objective was to assess tumor response to denosumab using three different imaging parameters in a prespecified analysis in patients with GCTB from two phase 2 studies.

**Methods:**

The studies enrolled adults and adolescents (skeletally mature and at least 12 years of age) with radiographically measurable GCTB that were given denosumab 120 mg every 4 weeks, with additional doses on days 8 and 15 of cycle 1. The proportion of patients with an objective tumor response was assessed using either Response Evaluation Criteria in Solid Tumors version 1.1 (RECIST), European Organisation for Research and Treatment of Cancer response criteria (positron emission tomography [PET] scan criteria), or inverse Choi density/size (ICDS) criteria. Target lesions were measured by computed tomography or magnetic resonance imaging (both studies), PET (study 2 only), or plain film radiograph (study 2 only).

**Results:**

Most patients (71.6%) had an objective tumor response by at least one response criteria. Per RECIST, 25.1% of patients had a response; per PET scan criteria, 96.2% had a response; per ICDS, 76.1% had a response. 68.5% had an objective tumor response ≥ 24 weeks. Using any criteria, crude incidence of response ranged from 56% (vertebrae/skull) to 91% (lung/soft tissue), and 98.2% had tumor control ≥ 24 weeks. Reduced PET avidity appeared to be an early sign of response to denosumab treatment.

**Conclusion:**

Modified PET scan criteria and ICDS criteria indicate that most patients show responses and higher benefit rates than modified RECIST, and therefore may be useful for early assessment of response to denosumab.

**Trial registration:**

ClinicalTrials.gov Clinical Trials Registry NCT00396279 (retrospectively registered November 6, 2006) and NCT00680992 (retrospectively registered May 20, 2008).

**Electronic supplementary material:**

The online version of this article (10.1186/s12957-018-1478-3) contains supplementary material, which is available to authorized users.

## Background

Giant cell tumor of bone (GCTB) is a histologically benign bone tumor composed of mononuclear stromal and multinucleated giant cells that exhibit osteoclastic activity, typically arising in the metaphyseal/epiphyseal portions of long bones [1, 2]. GCTB causes significant bone destruction, leading to pain, pathologic fracture, and impaired joint structure and functionality [3, 4]. Surgical resection is the primary curative method for GCTB; however, aggressive interventions, such as adjuvant therapy with liquid nitrogen or phenol, are often required to decrease morbidity, avoid amputation, and ensure adequate local control [4, 5]. Effective treatment options are limited for patients with lesions in locations not amenable to surgical resection [4], and local recurrence develops after several years in approximately 10–50% and 5% of patients after intralesional treatment or wide resection, respectively [5–8].

Constitutive activation of receptor activator of nuclear factor-kappa B (RANK) ligand maintains the osteolytic phenotype in GCTB [9, 10]. Denosumab (XGEVA^®^, Amgen Inc., Thousand Oaks, CA, USA), a RANK ligand inhibitor, is a fully human monoclonal antibody approved for the treatment of unresectable GCTB or when resection may result in severe morbidity. Denosumab treatment of GCTB prevents further tumor progression, reduces tumor size, reforms mineralized bone, and increases intralesional bone density [10, 11].

Radiologic assessment of tumor response in bone tumors presents unique challenges, and no uniform radiographic assessment criteria to date have been advanced to specifically assess response in GCTB. To address this challenge, our analysis combined imaging assessment techniques and captured response elements from three response evaluation measures widely employed in the assessment of change in tumor burden across a variety of tumor types, with modifications to tailor the response measures specifically to the unique properties of GCTB. Imaging records from two phase 2 clinical trials that supported denosumab registration [10, 11] were analyzed with three imaging parameters to measure the changes in lesion size and density, compare available radiographic parameters, and assess treatment response to denosumab in patients with GCTB.

## Methods

### Study design

This analysis used data pooled from two phase 2, open-label, single-arm, international, multicenter studies of denosumab [10, 11] in skeletally mature patients (≥ 12 years of age) with histologically confirmed GCTB and radiographically measurable disease. Key exclusion criteria included current use of alternative GCTB treatments (e.g., radiation, chemotherapy, embolization, or bisphosphonates). Study 1 [10] is complete; study 2 is ongoing [11]. In both studies, patients received 120 mg denosumab subcutaneously every 4 weeks, with additional loading doses on days 8 and 15 of the first treatment cycle (i.e., month 1). Patients received denosumab until disease progression and no clinical benefit, patient decision to withdraw from the study, or until complete tumor resection. In study 2 [11], patients with complete tumor resection received an additional six doses of denosumab after resection.

### Imaging assessments

**Fig. 1:**
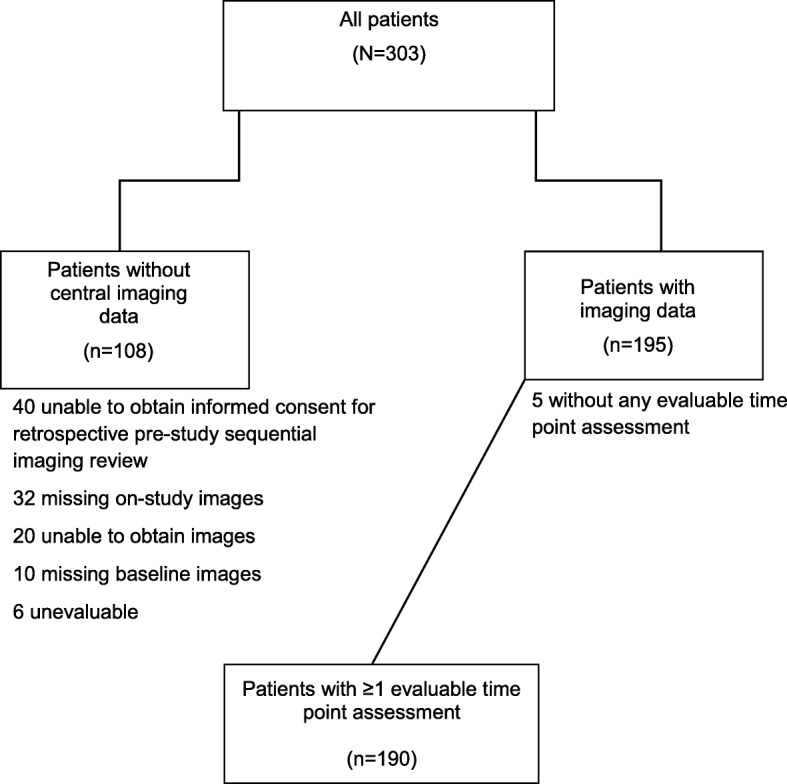
CONSORT diagram

Patients with ≥ 1 evaluable time point assessment were included in this analysis (Fig. 1). In study 1, computed tomography (CT) or magnetic resonance imaging (MRI) was required every 3 months [10], and in study 2, the imaging modality and frequency followed the local standard practice, which included plain film radiograph, CT, MRI, and 2-deoxy-2-[^18^F]fluoro-d-glucose positron emission tomography (^18^FDG-PET) [11]. Lesion images were retrospectively reviewed centrally by experienced bone radiologists blinded to investigator assessment. The central review was performed using a charter-specified, two-reader paradigm, with adjudication in case of interpretation discordance [11]. Key parameters and processes of the integrated, independent analysis of objective tumor response were agreed upon following consultation with regulatory authorities.

All available CT, MRI, and whole-body ^18^FDG-PET images were provided for the assessment of tumor response and disease progression using prespecified criteria (Table 1). Up to three response evaluation parameters were used to capture the unique anatomic and radiologic features of each lesion and response to treatment. These included criteria for modified Response Evaluation Criteria in Solid Tumors version 1.1 (RECIST), European Organisation for Research and Treatment of Cancer (EORTC; referred to as PET scan criteria), and inverse Choi density/size (ICDS) as outlined in Table 1 [12–14]. Postbaseline time points for assessment of tumor response, including the length of therapy by the patient, are summarized in Additional file 1: Figure S1.

**Table 1:** Response criteria

	Modified RECIST 1.1[13]	Modified EORTC [12]	ICDS [14]
CR	Disappearance of all target lesions; all target lymph nodes are < 10 mm in the short axis	Complete resolution of abnormal ^18^FDG uptake within the tumor volume of all target lesions to a level that is indistinguishable from surrounding normal tissue	Disappearance of all disease
PR	At least a 30% decrease in SLD using baseline SLD as a reference	Reduction of the sum of the SUV_max_ by ≥ 15–25% after 1 cycle and a decrease of ≥ 25% compared with baseline after > 1 treatment cycle	A decrease in size (%Δ Choi SLD) ≥ 10% or an increase in CT density > 15% compared with baseline, no new lesions, and no obvious progression of nonmeasurable disease
SD	Neither sufficient shrinkage of target lesions to qualify for PR nor sufficient increase to qualify for PD, taking as reference the nadir SLD	%ΔΣ SUV_max_ increased by < 25% or decreased by < 15% compared with baseline and no visible extent of ^18^FDG tumor uptake (> 20% in the longest dimension)	Does not meet the criteria for CR, PR, or PD; no symptomatic deterioration attributed to tumor progression
PD	At least a 20% increase in the SLD of target lesions, taking as reference the nadir SLD; in addition to the relative increase of 20% in SLD, the SLD must also demonstrate an absolute increase of ≥ 5 mm	%ΔΣ SUV_max_ increased by ≥ 25% compared with baseline scan, visible increase in the extent of ^18^FDG uptake (> 20% in the longest dimension) or the appearance of new ^18^FDG uptake in metastatic lesions	An increase in unidimensional tumor size (Choi SLD) ≥ 10% and does not meet the criteria for PR using CT density; any new lesions identified by CT/MRI; new intratumoral nodules or increase in the size of existing intratumoral nodules
UE	A target lesion present at baseline that subsequently became UE	^18^FDG-PET exam was unavailable or deemed UE;^a^ response will be UE unless unequivocal PD is determined on the basis of the evaluable target lesion	The CT/MRI exam is unavailable or deemed UE; if a target lesion is deemed UE by density and size measurement and the rules for PD do not apply, a response of CR, PR, or SD cannot be assigned for the time point and the response will be UE

*RECIST* Response Evaluation Criteria in Solid Tumors, *EORTC* European Organisation for Research and Treatment of Cancer, *ICDS* inverse Choi density/size, *CR* complete response, ^*18*^*FDG-PET* 2-deoxy-2- [^18^F]-fluorodeoxyglucose positron emission tomography, *PR* partial response, *SLD* sum of longest diameter, *SUV*_*max*_ maximum standardized uptake value, *SD* stable disease, *PD* progressive disease *CT* computed tomography, *MRI* magnetic resonance imaging, *UE* unevaluable

^a^The UE rate for this study was essentially 0

#### Statistics

Statistical analyses were descriptive in nature, and only summary statistics were presented. The analyses included the proportion of patients with an objective tumor response, time to first objective tumor response, duration of objective tumor response, and the proportions of patients with sustained (≥ 4, 12, and 24 weeks) objective tumor response and tumor control (complete response [CR], partial response [PR], or stable disease [SD]). Objective tumor response was defined as either CR or PR using any of the three tumor response evaluation criteria. The proportion of patients with an objective tumor response by baseline target lesion location and the percentage changes from baseline for lesion diameter and density were also summarized.

## Results

### Patients

Of the 303 patients, 190 (study 1 [*n* = 27] and study 2 [*n* = 163]) were included in this analysis. Of these, 187 had measurable anatomic lesion size evaluable by CT, 26 had functional imaging by ^18^FDG-PET, and 176 had CT-evaluable lesions, assessed for Hounsfield unit (HU) density and size, and were included in the RECIST, PET scan criteria, and ICDS evaluations, respectively.

Study 1 patients primarily had axial skeleton lesions not amenable to surgery with curative intent. Study 2 patients were divided into resectable lesions for which surgery could lead to significant morbidity (cohort 1) and unresectable tumors (cohort 2). All patients had radiographic evidence of active primary or recurrent GCTB within the previous year, with target lesions distributed across the disease spectrum; pelvis/sacrum (*n* = 61; 32%), lower extremities (*n* = 39; 21%), and lung (*n* = 38; 20%) were common target lesion sites. Most patients (70%) had prior GCTB resection/surgery, 20% had received prior bisphosphonates, and 20% had received prior radiotherapy (Table 2). Median (range) of time of patient participation was 13.4 months (1.7–48.9); patients received a median (range) of 16 doses (4–54) of denosumab. Baseline demographics and disease characteristics for patients without evaluable imaging analysis were similar to the population included in this analysis (Amgen Inc., data on file).

**Table 2:** Baseline demographics and disease characteristics

	Overall (*N* = 190)
Sex, *n* (%)	
Female	105 (55)
Male	85 (45)
Age, median (Q1, Q3), years	33 (26, 43)
ECOG performance status^a^, *n* (%)	
0	106 (56)
1	76 (40)
2	6 (3)
Previous treatment	
Resection/surgery	132 (70)
Bisphosphonates	38 (20)
Radiotherapy	37 (20)
Chemotherapy	21 (11)
GCTB disease type, *n* (%)	
Recurrent unresectable	92 (48)
Primary unresectable	43 (23)
Recurrent resectable	29 (15)
Primary resectable	26 (14)
Location of target lesion^b^, *n* (%)	
Pelvis/sacrum	61 (32)
Lower extremities	39 (21)
Lung	38 (20)
Spine	18 (10)
Upper extremities	17 (9)
Other^c^	11 (6)
Skull/neck	5 (3)
Missing	1 (1)

*Q* quartile, *ECOG* Eastern Cooperative Oncology Group, *GCTB* giant cell tumor of bone

^a^ECOG missing for two patients

^b^Based on case report form

^c^Includes other soft tissue and bone sites

Overall, 136/190 patients (71.6% [95% CI, 64.6–77.9%]) had an objective tumor response (CR or PR) by at least one response criteria. Per RECIST, 47/187 patients (25.1% [95% CI, 19.1–32.0%]) had a response; per PET scan criteria, 25/26 patients (96.2% [95% CI, 80.4–99.9%]) had a response; per ICDS, 134/176 patients (76.1% [95% CI, 69.1–82.2%]) had a response (Table 3). Using any response criteria, the median time to first objective tumor response (Kaplan-Meier estimate) was about 3 months per PET scan and ICDS criteria and was not estimable per RECIST. Overall, tumor responses were sustained; most patients (68.5%) had an objective tumor response for ≥ 24 weeks (Table 3). When analyzed by study and cohort, response rates were similar for PET scan criteria and ICDS (Table 3). Variations were observed when using RECIST, which showed a lower rate of response for study 1 (11%) than study 2 (28%). Within study 2, the response rates per RECIST were 32% and 17% for cohort 1 and cohort 2, respectively (Table 3). Similar results were observed for sustained objective tumor responses at weeks 4, 12, and 24 (Table 3).

**Table 3:** Objective tumor response results^a^

	Overall best response	RECIST 1.1	EORTC	ICDS
Proportion of responders, *n*/*N* (%)				
Overall	136/190 (71.6)	47/187 (25.1)	25/26 (96.2)	134/176 (76.1)
Study 1	20/27 (74.1)	3/27 (11.1)	15/16 (93.8)	18/23 (78.3)
Study 2	116/163 (71.2)	44/160 (27.5)	10/10 (100.0)	116/153 (75.8)
Cohort 1	76/114 (66.7)	36/113 (31.9)	4/4 (100.0)	76/105 (72.4)
Cohort 2	40/49 (81.6)	8/47 (17.0)	6/6 (100.0)	40/48 (83.3)
Median time to first OTR, months (95% CI)^b^	3.1 (2.89–3.65)	NE (20.9–NE)	2.7 (1.64–2.79)	3.0 (2.79–3.48)
Patients with sustained OTR, *n*/*N* (%)				
Overall				
≥ 4 weeks	102/153 (66.7)	32/150 (21.3)	18/20 (90.0)	101/143 (70.6)
≥ 12 weeks	98/144 (68.1)	32/141 (22.7)	16/17 (94.1)	97/135 (71.9)
≥ 24 weeks	76/111 (68.5)	26/109 (23.9)	11/12 (91.7)	76/102 (74.5)
Study 1				
≥ 4 weeks	15/24 (62.5)	2/24 (8.3)	11/13 (84.6)	13/20 (65.0)
≥ 12 weeks	14/20 (70.0)	2/20 (10.0)	10/11 (90.9)	13/17 (76.5)
≥ 24 weeks	12/17 (70.6)	2/17 (11.8)	8/9 (88.9)	12/14 (85.7)
Study 2				
≥ 4 weeks	87/129 (67.4)	30/126 (23.8)	7/7 (100.0)	88/123 (71.5)
≥ 12 weeks	84/124 (67.7)	30/121 (24.8)	6/6 (100.0)	84/118 (71.2)
≥ 24 weeks	64/94 (68.1)	24/92 (26.1)	3/3 (100.0)	64/88 (72.7)
Cohort 1				
≥ 4 weeks	59/91 (64.8)	25/90 (27.8)	3/3 (100.0)	60/85 (70.6)
≥ 12 weeks	56/87 (64.4)	25/86 (29.1)	3/3 (100.0)	57/81 (70.4)
≥ 24 weeks	49/73 (67.1)	22/73 (30.1)	2/2 (100.0)	50/67 (74.6)
Cohort 2				
≥ 4 weeks	28/38 (73.7)	5/36 (13.9)	4/4 (100.0)	28/38 (73.7)
≥ 12 weeks	28/37 (75.7)	5/35 (14.3)	3/3 (100.0)	27/37 (73.0)
≥ 24 weeks	15/21 (71.4)	2/19 (10.5)	1/1 (100.0)	14/21 (66.7)
Patients with tumor control^c^, %				
≥ 4 weeks	148/153 (96.7)	145/150 (96.7)	19/20 (95.0)	139/143 (97.2)
≥ 12 weeks	139/144 (96.5)	137/141 (97.2)	17/17 (100.0)	131/135 (97.0)
≥ 24 weeks	109/111 (98.2)	108/109 (99.1)	12/12 (100.0)	101/102 (99.0)

*RECIST* Response Evaluation Criteria in Solid Tumors, *EORTC* European Organisation for Research and Treatment of Cancer, *ICDS* inverse Choi density/size; *NE* not estimable, *OTR* objective tumor response

^a^Patients with at least one evaluable time point assessment

^b^Kaplan-Meier estimate

^c^Defined as CR + PR + SD

**Fig. 2:**
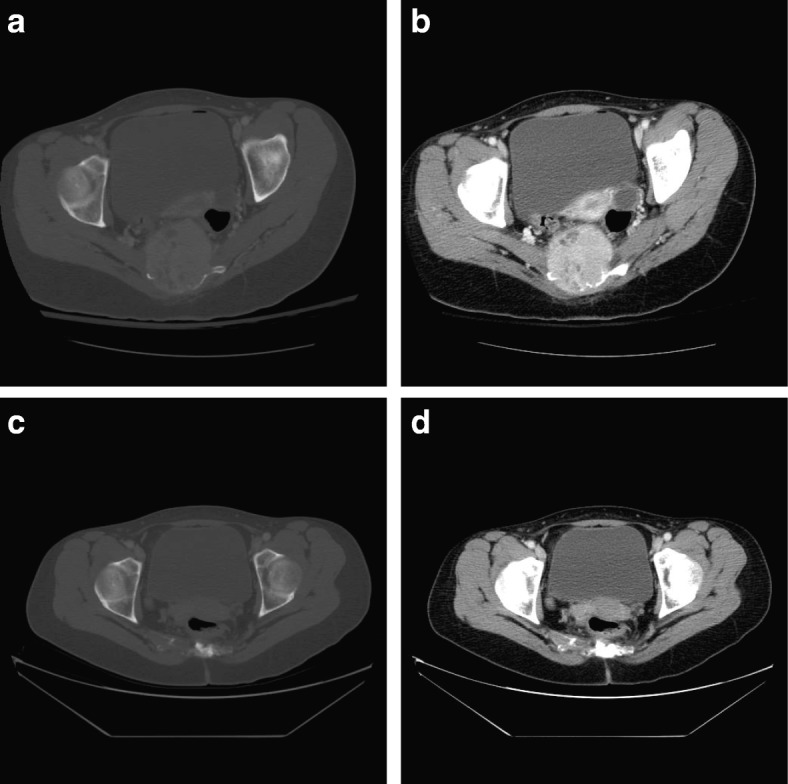
Sacral GCTB before and after treatment with denosumab. **a** Bone window and **b** soft tissue window pretreatment CT scan from August 14, 2009, through the level of the upper hip joints. There is extensive bone destruction and a large soft tissue mass that displaces the rectum. **c** Bone window and **d** soft tissue window CT repeat scan on December 12, 2013 (about 4 years and 4 months later) following treatment with denosumab (about 3 years and 10 months; first dose on January 21, 2010, and last dose on November 21, 2013). The soft tissue mass is now negligible, and the bone is reconstituting. *CT* computed tomography; *GCTB* giant cell tumor of bone

Objective tumor response by target lesion location showed that the crude incidences of response (95% CI) using any criteria were 14/24 (58.3% [36.6–77.9%]) for pelvis, 22/37 (59.5% [42.1–75.2%]) for sacrum, 32/40 (80.0% [64.4–90.9%]) for lower extremity, 39/43 (90.7% [77.9–97.4%]) for lung/soft tissue, 15/20 (75.0% [50.9–91.3%]) for upper extremity, and 14/25 (56.0% [34.9–75.6%]) for vertebrae/skull. Figure 2 shows CT images before and after denosumab treatment in a patient with sacral GCTB. Tumor control ≥ 24 weeks was observed in 98.2% of patients using any criteria; similar rates were observed for the other response criteria (Table 3).

Median (range) lesion size was 62.5 mm (10–283), consistent with the advanced disease in the study population (Table 4). Anatomic extent, measured by longest diameter (LD), demonstrated that the greatest percentage decreases in size occurred ≤ 3 months on-study and were consistent and sustained. Considering the best percentage change in LD, arrangement in increasing order of degree of response per the ICDS evaluation (Additional file 1: Figure S2a) revealed a group of patients that did not respond to therapy, with an LD increase ≥ 10% (*n* = 4, 2%); a second group of patients with SD and LD changes ± 10% (*n* = 76, 43%); and a third group of patients with an LD decreases ≥ 10% (*n* = 95, 54%). For responders (defined by ≥ 10% reduction in tumor size; Table 1) with a measurable decrease in LD, there was an evenly graded distribution of best LD reduction ranging from 11 to > 70%.

**Table 4:** Baseline LD and SUV_max_ summary in patients with ≥ 1 evaluable time point assessment of ^**18**^FDG-PET avidity

	*n*	Mean	SD	Min	Q1	Median	Q3	Max
LD, mm	174	68.4	40.8	10.0	38.0	62.5	91.0	283.0
SUV_max_	26	11.1	4.7	3.8	7.9	10.6	13.6	21.6

^*18*^*FDG-PET* 2-deoxy-2-[^18^F]-fluorodeoxyglucose positron emission tomography, *LD* longest diameter, *max* maximum, *min* minimum, *Q* quartile, *SUV*_*max*_ maximum standardized uptake value

Using HU density as a response parameter, the best percentage change in density for target lesions showed that 99 of 124 patients (80%) had ≥ 15% increase and 25 patients (20%) had < 15% increase (Additional file 1: Figure S2b); 15% is the density cutoff for the response per Choi gastrointestinal stromal tumor (GIST) criteria [14]. HU evaluation showed that percentage increases in tumor density ≤ 6 months on-study were consistent and sustained; mean HU values rarely decreased once increases were observed, with medians of 93 and 108 at postbaseline time point assessments 1 and 2, respectively. Time point assessments were ≥ 24 weeks apart [11].

At baseline, the mean (SD) maximum standardized uptake value (SUV_max_) of ^18^FDG-PET in 26 patients using PET scan criteria was 11.1 (4.7), indicative of high metabolic activity in GCTB lesions before denosumab treatment (Table 4). Almost 100% of lesions showed a rapid reduction in ^18^FDG-PET avidity at the earliest time point assessment (Table 3). PET responsiveness did not appear to vary with lesion location. Reduction in ^18^FDG-PET avidity therefore appeared to be an early and universal sign of response to denosumab treatment.

## Discussion

We observed impressive tumor control rates, with nearly all patients with GCTB showing sustained tumor control for ≥ 24 weeks, using any of the response criteria. Increases in lesion density by HU likely reflected the pharmacodynamic response to denosumab treatment (i.e., suppression of osteolysis and increased formation of dense fibro-osseous tissue and/or woven bone [9]). This clinical benefit allows patients to defer or downstage their planned surgical procedure when surgical resection is likely to result in severe morbidity [15]. In contrast, a purely size-based evaluation using RECIST is potentially insensitive in assessing response in bone lesions with a mixed osteolytic and expanding soft tissue component; the size of GCTB tumors changes little with targeted therapies. Therefore, an inverse modification of the ICDS was used to evaluate both GCTB density and size; either a decrease in size or an increase in density was considered a response to treatment. In GCTB, decreases in tumor size per LD are believed to reflect cytoreduction, in alignment with RECIST principles for solid tumor assessment. The kinetics of GCTB responses to denosumab therapy showed rapid cytoreduction that peaked by 3 months and was maintained thereafter, with responses of ≥ 24 weeks in nearly all patients. The Choi criteria were developed to monitor response in a soft tissue sarcoma undergoing targeted therapy where tumor cell viability and radiological size reduction may be uncoupled during the response to treatment [14]. Analogous to GIST, in the setting of GCTB, we believe that the ICDS criteria used in the present study perform as pharmacodynamic markers of effect and may offer an advantage to conventional RECIST.

In our study, patients had unresectable tumors or tumors requiring highly invasive or disabling surgery in an attempt to achieve surgical cure; therefore, there was a large number of pelvic, spine, and pulmonary lesions that complicated radiographic evaluation of response. Using ICDS, four patients had a ≥ 10% increase in LD, two of whom sustained increases in tumor size after study enrollment but before administration of denosumab. These two patients experienced sustained disease control lasting several months while receiving denosumab continuously, and for one patient, 12 additional months of disease control following discontinuation of denosumab. The remaining two patients had atypical GCTB. One had multiostotic and metastatic GCTB with lesions in the pelvis, rib, and lung at study entry and received denosumab for 8 months before being lost to follow-up. Multiostotic GCTB accounts for < 1% of all GCTB and has a different clinical presentation than solitary lesions; typically, patients are younger, suggesting a germ-line component that confers susceptibility to the disease [16–21]. The other patient with atypical GCTB with an increased tumor size had a clinically aggressive disease with ten previous attempts at surgical resection before enrollment. While these patients met all histological entry criteria and had pathologically confirmed GCTB, it remains unclear whether their atypical courses before and during denosumab treatment suggest an aggressive clinical variant of classical GCTB or an alternative diagnosis. Because true nonresponse to denosumab in GCTB is rare, patients with nonresponse may deserve more comprehensive sampling for histological disease assessment. The best percentage change in density for target lesions in the ICDS evaluation showed that 80% of the 124 patients evaluable for density had a ≥ 15% increase in density, reflecting the desired outcome of denosumab therapy.

Our results confirm and extend findings reported in a smaller study [22] where 88% patients (*n* = 17) had an objective tumor response using any response criteria after denosumab treatment (median duration of 13.1 months). In the present study, the proportions of patients with an objective tumor response were 35% per RECIST, 82% per PET scan criteria, and 71% per ICDS criteria (size/density). The median time to objective tumor response using any of the response criteria was 3.0 months (95% CI, 2.9–3.1). The benefit of denosumab in GCTB has already been established [10, 11]; our results provide clinicians with additional information on imaging and monitoring patients with GCTB treated with denosumab.

The single-arm study design limits our analysis; however, the central independent review of images was conducted to minimize this limitation. Furthermore, this study had a large number of unevaluable patients, there was no protocol-defined imaging schedule or methodology (which is standard for this type of study), and only a few PET scans were done, as PET was optional. We also limited our definition of sustained tumor control to a time frame of 24 weeks, which may be considered short by some clinicians; however, there are no well-established tumor response criteria for patients with GCTB [22]. We also did not examine any association between response and extent of prior treatment or other factors. The retrospective nature of this analysis made obtaining historical images difficult.

There are inherent limitations associated with using RECIST alone for assessment of denosumab response in GCTB because of the sometimes modest reduction in tumor size despite clinical benefit. Reduction in ^18^FDG-PET avidity predicted a favorable tumor response and sustained tumor control with denosumab treatment. Given the rarity of denosumab refractoriness in typical GCTB, new or continued high SUV_max_ levels while on denosumab should alert clinicians to the possibility of an aggressive clinical variant or an alternate diagnosis such as sarcoma.

Our data do not suggest an increase in the risk of osteosarcoma following denosumab treatment. There are recent case studies of patients with GCTB treated with denosumab who have developed osteosarcoma [23, 24]; three patients were diagnosed with osteosarcoma during denosumab treatment in primary reports of the studies used for our analysis [10, 11]. Patients with GCTB are at higher risk for developing osteosarcoma than the general population, with approximately 2–5% of patients developing secondary sarcoma following radiotherapy or surgical resection [25–27]. There also remains the previously reported, equally difficult task of identifying patients with small foci of sarcomatous change within the large field of otherwise benign-appearing GCTB [28]. The incidence of pathologic fracture is up to 30% in patients with GCTB; data to date do not indicate an increased rate with denosumab [8, 29, 30].

## Conclusions

Modified PET scan criteria and ICDS criteria showed responses in most patients in our analysis, indicating a substantially higher benefit rate compared to that assessed by modified RECIST. PET or CT with ICDS provided an early indication of treatment response. Moreover, all response criteria indicated tumor control ≥ 24 weeks to denosumab. Loss of ^18^FDG-PET avidity may have a dual role in both predicting long-term disease control and offering clinicians some reassurance that there is not a focus of sarcoma with the GCTB lesion, which would likely remain ^18^FDG-PET avid despite denosumab treatment. Further research is required to determine the appropriate imaging technique to be used longitudinally in a given patient, although many practitioners favor a combination of plain radiographs and CT. Regardless of the modality used, careful evaluation of nonresponders is necessary.

## Additional file

Additional file 1: **Figure S1.** Postbaseline time point assessments for tumor response by study for patients with ≥ 1 evaluable time point assessment. Per protocol, the sites were instructed to perform CT or MRI scans of the lesion at baseline and quarterly during the treatment period. ^18^FDG-PET scans were performed at the discretion of the investigator. Because this was a retrospective, independent image review, no specific acquisition parameters were provided. Sites were instructed to use their standard acquisition parameters for CT, MRI, and ^18^FDG-PET. Consistent use of the imaging modalities, parameters, and contrast was recommended for reproducibility. *CT* computed tomography, ^*18*^*FDG‑PET* 2-deoxy-2-[^18^F] fluoro-D-glucose positron emission tomography; *MRI* magnetic resonance imaging. **Figure S2.** (a) Best percentage change in SLD for target lesions in the ICDS evaluation and (b) best percentage change in density for target lesions in the ICDS evaluation. *ICDS* inverse Choi density/size; *LD* longest diameter; *SLD* sum of longest diameter. (DOCX 233 kb)

## Abbreviations

^18^FDG-PET: 2-Deoxy-2-[^18^F] fluoro-d-glucose positron emission tomography
CR: Complete response
CT: Computed tomography
EORTC: European Organisation for Research and Treatment of Cancer
GCTB: Giant cell tumor of bone
GIST: Gastrointestinal stromal tumor
HU: Hounsfield unit
ICDS: Inverse Choi density/size
LD: Longest diameter
MRI: Magnetic resonance imaging
PET: Positron emission tomography
PR: Partial response
RANK: Receptor activator of nuclear factor-kappa B
RECIST: Response Evaluation Criteria in Solid Tumors version 1.1
SD: Stable disease
SUV_max_: Maximum standardized uptake value

## References

[1] Turcotte RE (2006). Giant cell tumor of bone. Orthop Clin North Am.

[2] Futamura N, Urakawa H, Tsukushi S, Arai E, Kozawa E, Ishiguro N, Nishida Y (2016). Giant cell tumor of bone arising in long bones possibly originates from the metaphyseal region. Oncol Lett.

[3] Enneking WF. A system of staging musculoskeletal neoplasms. Clin Orthop Relat Res. 1986;209:9–24. 3456859

[4] Szendrői Miklós (2014). Giant-Cell Tumour of Bone (GCT). European Surgical Orthopaedics and Traumatology.

[5] Klenke FM, Wenger DE, Inwards CY, Rose PS, Sim FH (2011). Recurrent giant cell tumor of long bones: analysis of surgical management. Clin Orthop Relat Res.

[6] Szendroi M (2004). Giant-cell tumour of bone. J Bone Joint Surg Br.

[7] Arbeitsgemeinschaft K, Becker WT, Dohle J, Bernd L, Braun A, Cserhati M, Enderle A, Hovy L, Matejovsky Z, Szendroi M (2008). Local recurrence of giant cell tumor of bone after intralesional treatment with and without adjuvant therapy. J Bone Joint Surg Am.

[8] Campanacci M, Baldini N, Boriani S, Sudanese A (1987). Giant-cell tumor of bone. J Bone Joint Surg Am.

[9] Branstetter DG, Nelson SD, Manivel JC, Blay JY, Chawla S, Thomas DM, Jun S, Jacobs I (2012). Denosumab induces tumor reduction and bone formation in patients with giant-cell tumor of bone. Clin Cancer Res.

[10] Thomas D, Henshaw R, Skubitz K, Chawla S, Staddon A, Blay JY, Roudier M, Smith J, Ye Z, Sohn W (2010). Denosumab in patients with giant-cell tumour of bone: an open-label, phase 2 study. Lancet Oncol.

[11] Chawla S, Henshaw R, Seeger L, Choy E, Blay JY, Ferrari S, Kroep J, Grimer R, Reichardt P, Rutkowski P (2013). Safety and efficacy of denosumab for adults and skeletally mature adolescents with giant cell tumour of bone: interim analysis of an open-label, parallel-group, phase 2 study. Lancet Oncol.

[12] Young H, Baum R, Cremerius U, Herholz K, Hoekstra O, Lammertsma AA, Pruim J, Price P (1999). Measurement of clinical and subclinical tumour response using [18F]-fluorodeoxyglucose and positron emission tomography: review and 1999 EORTC recommendations. European Organization for Research and Treatment of Cancer (EORTC) PET Study Group. Eur J Cancer.

[13] Eisenhauer EA, Therasse P, Bogaerts J, Schwartz LH, Sargent D, Ford R, Dancey J, Arbuck S, Gwyther S, Mooney M (2009). New response evaluation criteria in solid tumours: revised RECIST guideline (version 1.1). Eur J Cancer.

[14] Choi H, Charnsangavej C, Faria SC, Macapinlac HA, Burgess MA, Patel SR, Chen LL, Podoloff DA, Benjamin RS (2007). Correlation of computed tomography and positron emission tomography in patients with metastatic gastrointestinal stromal tumor treated at a single institution with imatinib mesylate: proposal of new computed tomography response criteria. J Clin Oncol.

[15] Rutkowski P, Ferrari S, Grimer RJ, Stalley PD, Dijkstra SP, Pienkowski A, Vaz G, Wunder JS, Seeger LL, Feng A (2015). Surgical downstaging in an open-label phase II trial of denosumab in patients with giant cell tumor of bone. Ann Surg Oncol.

[16] Wu KK, Ross PM, Mitchell DC, Sprague HH. Evolution of a case of multicentric giant cell tumor over a 23-year period. Clin Orthop Relat Res. 1986;213:279–88. 3780103

[17] Sim FH, Dahlin DC, Beabout JW (1977). Multicentric giant-cell tumor of bone. J Bone Joint Surg Am.

[18] Tornberg DN, Dick HM, Johnston AD (1975). Multicentric giant-cell tumors in the long bones. A case report. J Bone Joint Surg Am.

[19] Hindman BW, Seeger LL, Stanley P, Forrester DM, Schwinn CP, Tan SZ (1994). Multicentric giant cell tumor: report of five new cases. Skelet Radiol.

[20] Hoch B, Inwards C, Sundaram M, Rosenberg AE (2006). Multicentric giant cell tumor of bone. Clinicopathologic analysis of thirty cases. J Bone Joint Surg Am.

[21] Wirbel R, Blumler F, Lommel D, Syre G, Krenn V (2013). Multicentric giant cell tumor of bone: synchronous and metachronous presentation. Case Rep Orthop.

[22] Ueda T, Morioka H, Nishida Y, Kakunaga S, Tsuchiya H, Matsumoto Y, Asami Y, Inoue T, Yoneda T (2015). Objective tumor response to denosumab in patients with giant cell tumor of bone: a multicenter phase II trial. Ann Oncol.

[23] Aponte-Tinao LA, Piuzzi NS, Roitman P, Farfalli GL (2015). A high-grade sarcoma arising in a patient with recurrent benign giant cell tumor of the proximal tibia while receiving treatment with denosumab. Clin Orthop Relat Res.

[24] Broehm CJ, Garbrecht EL, Wood J, Bocklage T (2015). Two cases of sarcoma arising in giant cell tumor of bone treated with denosumab. Case Rep Med.

[25] Bertoni F, Bacchini P, Staals EL (2003). Malignancy in giant cell tumor of bone. Cancer.

[26] Sanerkin NG (1980). Malignancy, aggressiveness, and recurrence in giant cell tumor of bone. Cancer.

[27] McGrath PJ (1972). Giant-cell tumour of bone: an analysis of fifty-two cases. J Bone Joint Surg Br.

[28] Hefti FL, Gachter A, Remagen W, Nidecker A (1992). Recurrent giant-cell tumor with metaplasia and malignant change, not associated with radiotherapy. A case report. J Bone Joint Surg Am.

[29] Turcotte Robert E., Wunder Jay S., Isler Marc H., Bell Robert S., Schachar Norman, Masri Bassam A., Moreau Guy, Davis Aileen M. (2002). Giant Cell Tumor of Long Bone: A Canadian Sarcoma Group Study. Clinical Orthopaedics and Related Research.

[30] Sung HW, Kuo DP, Shu WP, Chai YB, Liu CC, Li SM (1982). Giant-cell tumor of bone: analysis of two hundred and eight cases in Chinese patients. J Bone Joint Surg Am.

