# Supplementary material for: Assessment of denosumab treatment effects and imaging response in patients with giant cell tumor of bone
Source: World J Surg Oncol. 2018 Sep 19;16:191. doi: 10.1186/s12957-018-1478-3 (PMC6146657; doi:10.1186/s12957-018-1478-3)
Supplement: Supplementary file 1 — Figure S1. Postbaseline time point assessments for tumor response by study for patients with ≥ 1 evaluable time point assessment. Per protocol, the sites were instructed to perform CT or MRI scans of the lesion at baseline and quarterly during the treatment period. 18FDG-PET scans were performed at the discretion of the investigator. Because this was a retrospective, independent image review, no specific acquisition parameters were provided. Sites were instructed to use their standard acquisition parameters for CT, MRI, and 18FDG-PET. Consistent use of the imaging modalities, parameters, and contrast was recommended for reproducibility. CT computed tomography, 18FDG‑PET 2-deoxy-2-[18F] fluoro-D-glucose positron emission tomography; MRI magnetic resonance imaging. Figure S2. (a) Best percentage change in SLD for target lesions in the ICDS evaluation and (b) best percentage change in density for target lesions in the ICDS evaluation. ICDS inverse Choi density/size; LD longest diameter; SLD sum of longest diameter. (DOCX 233 kb) [file 12957_2018_1478_MOESM1_ESM.docx]

**Additional File 1**

# Additional Figure Legends

**Figure S1.** Postbaseline time point assessments for tumor response by study for patients with ≥1 evaluable time point assessment. Per protocol, the sites were instructed to perform CT or MRI scans of the lesion at baseline and quarterly during the treatment period. ^18^FDG-PET scans were performed at the discretion of the investigator. Because this was a retrospective, independent image review, no specific acquisition parameters were provided. Sites were instructed to use their standard acquisition parameters for CT, MRI, and ^18^FDG-PET. Consistent use of the imaging modalities, parameters, and contrast was recommended for reproducibility. *CT* computed tomography, *^18^FDG‑PET* 2-deoxy-2-[18F] fluoro-D-glucose positron emission tomography, *MRI* magnetic resonance imaging.

**Figure S2*.*** (a) Best percentage change in SLD for target lesions in the ICDS evaluation and (b) best percentage change in density for target lesions in the ICDS evaluation. *ICDS* Inverse Choi Density/Size; *LD* longest diameter; *SLD* sum of longest diameter.

# Figure S1.

**Study 1 (N=27) Study 2 (N=163)**

**
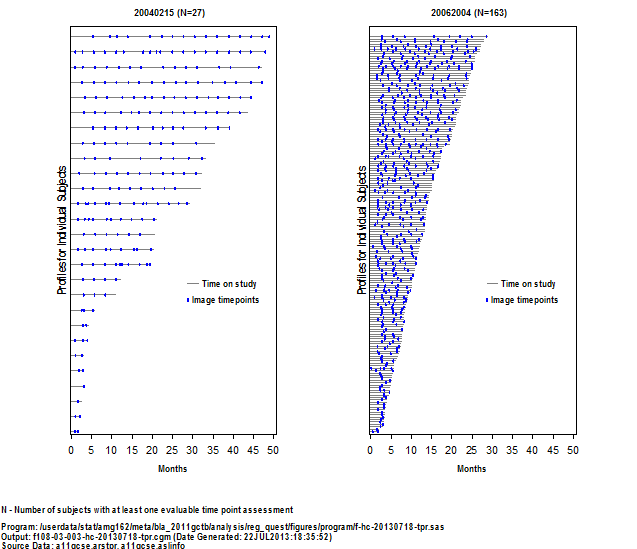
**

**Figure S2.**

**a**

**b**
